# Microevolution of Duplications and Deletions and Their Impact on Gene Expression in the Nematode *Pristionchus pacificus*

**DOI:** 10.1371/journal.pone.0131136

**Published:** 2015-06-30

**Authors:** Praveen Baskaran, Christian Rödelsperger

**Affiliations:** Department for Evolutionary Biology, Max Planck Institute for Developmental Biology, Tübingen, Germany; Inserm U869, FRANCE

## Abstract

The evolution of diversity across the animal kingdom has been accompanied by tremendous gene loss and gain. While comparative genomics has been fruitful to characterize differences in gene content across highly diverged species, little is known about the microevolution of structural variations that cause these differences in the first place. In order to investigate the genomic impact of structural variations, we made use of genomic and transcriptomic data from the nematode *Pristionchus pacificus*, which has been established as a satellite model to *Caenorhabditis elegans* for comparative biology. We exploit the fact that *P. pacificus* is a highly diverse species for which various genomic data including the draft genome of a sister species *P. exspectatus* is available. Based on resequencing coverage data for two natural isolates we identified large (> 2kb) deletions and duplications relative to the reference strain. By restriction to completely syntenic regions between *P. pacificus* and *P. exspectatus*, we were able to polarize the comparison and to assess the impact of structural variations on expression levels. We found that while loss of genes correlates with lack of expression, duplication of genes has virtually no effect on gene expression. Further investigating expression of individual copies at sites that segregate between the duplicates, we found in the majority of cases only one of the copies to be expressed. Nevertheless, we still find that certain gene classes are strongly depleted in deletions as well as duplications, suggesting evolutionary constraint acting on synteny. In summary, our results are consistent with a model, where most structural variations are either deleterious or neutral and provide first insights into the microevolution of structural variations in the *P. pacificus* genome.

## Introduction

The development of multicellular organisms is coordinated by gene regulatory networks that ensure correct spatio-temporal patterns of gene expression. Thus gene dosage is expected to be tightly controlled and any kind of perturbation is highly likely to be deleterious. Nevertheless, the sequencing of hundreds of genomes throughout the animal kingdom, has revealed a tremendous amount of gene loss and gain during evolution of metazoan life [1–3].

One fundamental process that can cause loss and gain of genes at a microevolutionary level, are structural variations (SVs). While the changes in gene repertoires have extensively been documented in several comparative genomic studies at a cross-species level [4–6], only recently population-scale microarray and sequencing projects, have shown the tremendous impact of SVs as they affect larger parts of genomes than single nucleotide variation [7–11]. We have recently employed whole-genome sequencing of 104 natural isolates of the nematode *Pristionchus pacificus* to characterize patterns of genetic diversity at the single nucleotide level [12]. *P. pacificus* has been introduced as satellite model organism for comparative studies of development [13], feeding behavior [14], ecology [15], and population genetics [12] with the classic nematode model organism *Caenorhabditis elegans*. Recent population genomic analysis have revealed several lines of evidence for a prominent role of purifying selection including background selection, shaping genetic diversity across the genome of *P. pacificus*. This indicates that deleterious mutations and also linked neutral variants are rapidly purged from populations by purifying selection [12, 16]. While our previous analyses have mostly focused on patterns of single nucleotide variants, we would like to extend these studies by investigating the distribution and effect of SVs, which we define in this study as large (≥ 2kb) deletions and duplications. Incorporating the genome of a closely related sister species *P. exspectatus* allows us to polarize deletion and duplication events and to correctly interpret them in an evolutionary context. In addition, we use available transcriptome data, to assess the effect of deletion and duplication events on gene expression levels, because to our knowledge only few studies have combined the investigation of gene loss and gain with measurements of gene expression [17–19]. Naively, we would expect loss of expression in case of deletions and an increase by a factor of two in case of duplications. Open questions are, whether SV detection methods based on read coverage are accurate enough to identify gene loss and gain events and whether the effect of SVs on transcriptome profiles is so strong that it needs to be accounted for when doing differential gene expression analysis across strains.

In this study, we show that the combination of genomic and transcriptomic data together with the draft assembly of a sister species is strong enough to detect substantial signal of SVs on the genomic and transcriptomic level. The depletion of SVs among highly conserved genes, and the loss of expression evidence in most predicted deletions are the strongest arguments in favour for the validity of our employed methods. Interestingly, we find that our naive expectation concerning duplications is not fulfilled and by tracing the expression levels of sites that segregate between two copies, we find that most investigated genes exhibit strongly biased expression. In summary these findings highlight the either deleterious or neutral nature of detected deletions and duplications in the *P. pacificus* genome.

## Materials and Methods

### Analysis of raw sequencing data

Genomic and transcriptomic libraries were prepared as described previously [12, 13] and were sequenced as 100bp paired end reads on the Illumina HiSeq 2000 platform. Raw alignments of genomic reads for the three strains PS312 (16 million reads, 16X mean coverage after alignment), RS5200 (12 × 10^6^ reads, 12X), and RS5410 (8 × 10^6^ reads, 8X) were taken from the data set by Rödelsperger et al. [12]. In summary, these alignments were produced after quality trimming, alignment to the *P. pacificus* Hybrid1 assembly with the program stampy (v1.0.12) [20], duplicate read removal using samtools (v0.1–18) [21], and local realignment with the software GATK (v2.1–13) [22] (see [12] for further details).

Raw alignments of RNA-seq data for the three strains PS312 (19 × 10^6^ reads), RS5200 (16 × 10^6^ reads), and RS5410 (21 × 10^6^ reads) were taken from the data set by Ragsdale and Müller et al. [13]. These alignments were generated by the software Tophat v.2.0.3 which was run with default options and using the *P. pacificus* Hybrid1 assembly as reference. We used the program Cufflinks (v2.0.1) [23], to quantify the expression levels of genes (version TAU [24]) as fragments per kilobase transcript per million fragments sequenced (FPKM) and the accompanying cuffdiff program for differential expression analysis.

### Identification of deletions and duplications

In order to predict duplications and deletions that are sufficiently large to affect complete genes, we applied a differential read coverage approach, implemented in the software cnv-seq [25]. cnv-seq predicts deletion and duplication events by sliding a window across the genome and testing for significant differences in read coverage based on a Poisson model. We explicitly selected a differential approach which considers resequencing data of the reference strain PS312 as control sample, because this has the advantage, that false predictions due to assembly errors are minimized because they should equally affect the reference sample. The resulting predictions were evaluated previously by comparison with PCR amplification experiments for three genes in 24 strains [12, 26], showing perfect agreement between both methods. For example, our predictions based on read coverage recapitulate the finding of Mayer et al. showing the absence of one member (cel-3) of a gene family of horizonatally transferred cellulases from the strain RS5200 [26]. Moreover, one duplication in the reference strain PS312 that was identified by cnv-seq, was recently confirmed by inverse PCR and the corresponding duplicated gene was shown to inhibit dauer formation in a dose dependent manner [27]. To further improve the overall reliability of predictions for our two strains of interest, we generated a test set of manually classified true and false positive SVs, evaluated the performance depending on different parameters, and chose a combination of p-value and fold change thresholds that appeared to us as optimal.

**Fig 1:**
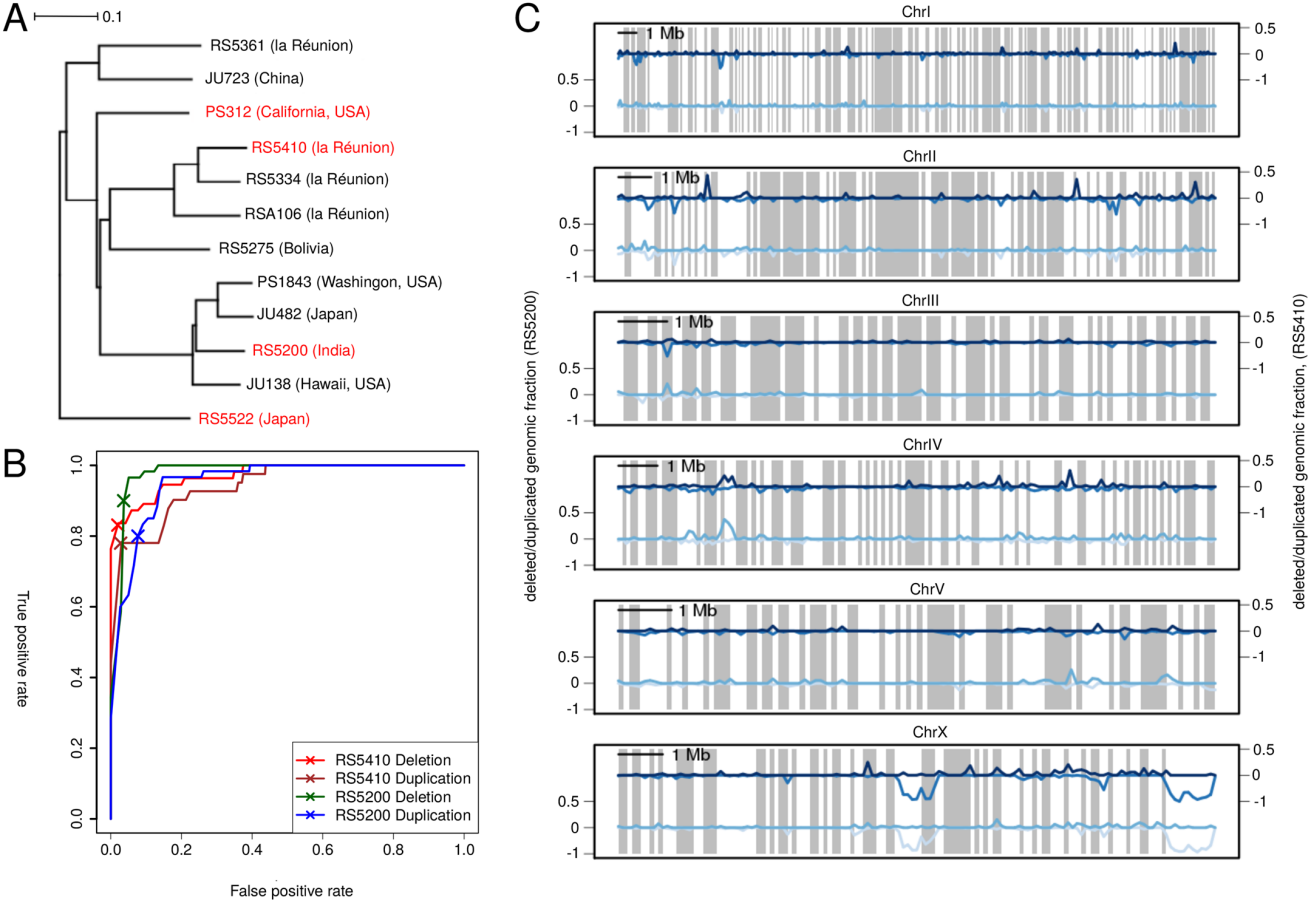
Identification of duplications and deletions in the *P. pacificus* strains. A) General phylogeny of *P. pacificus* strains and the sister species *P. exspectatus* (RS5522). Strains and species that are used in this study are colored in red. The tree was generated by building a neighbor joining tree on Hamming distances based on roughly 450,000 parsimony informative sites (singletons excluded). Excluding singletons leads to a vast underestimation of distances between strains and species but should increase the robustness of the tree topology. All internal nodes showed a perfect bootstrap support of 100/100. B) True and false positive rate for SV calls for various p-value and fold change cutoffs of the program cnv-seq. The evaluation is based on 100 manually classified SV calls per strain per SV type and final cutoff combinations were chosen subjectively. C) The graphs show the fraction of genomic sequence within non-overlapping 100kb windows, predicted to be duplicated (positive values) and deleted (negative values) relative to the reference strain PS312. Contigs were concatenated using the genetic map of *P. pacificus* to produce chromosome-scale plots [36]. Gray boxes indicate conserved syntenic regions with the sister species *P. exspectatus*. While duplications exhibit a much more even distribution, we identified two almost megabase sized regions with high fraction of missing sequence on the X chromosome.

More precisely, we selected randomly 100 duplications and deletions for each strain, for which we visualised alignments in the Integrative Genomics viewer [28] and manually classified each predicted structural variation as true positive or false positive. Thereby, we evaluated the predicted SVs based on additional features: coverage of the surrounding areas should be different even within the strain of interest, apparent heterozygous variant calls support the presence of a duplication, unusually high read coverage or fragmented islands of coverage in the reference sample indicate towards assembly problems. We then performed a receiver operating characteristic to examine the quality of predictions depending on varying thresholds for p-value (*P*) and log2 fold change (Fc) in read coverage (Fig 1B) and we finally arbitrarily chose a combination of thresholds that appeared to us as a good tradeoff between true and false positives. The chosen values were Fc = 1.01 and *P* < 10^−15^ for RS5410 duplications, Fc = -1.66 and *P* < 10^−5^ for RS5410 deletions, Fc = 0.74 and *P* < 10^−15^ for RS5200 duplications, Fc = -1.63 and *P* < 10^−5^ for RS5200 deletions. These values correspond to a true and false positive rate of 83% and 2% for deletion and 78% and 2.8% for duplications in RS5410 and 90% and 3.6% for deletion and 80% and 7.7% for duplications in RS5200. In order to generate a set of duplications with precise information of breakpoints, we applied a split read approach as implemented in the software pindel [29], which identified 183 tandem duplications (> 2kb) in RS5200 and 117 in RS5410.

### Identification of perfect collinearity with *P. exspectatus*

For polarization of duplications and deletions, we restricted the analysis to conserved syntenic regions between the reference strain and the sister species *P. exspectatus*. We defined conserved syntenic regions using the program cyntenator [30]. We used a sufficiently high gap and mismatch penalties (-gap -1000 -mis -1500) to ensure that gene orders between both genomes is the same (perfectly collinear). In addition we requested each gene to be represented only once within any collinear block (-coverage 1), set the minimal gene order alignment length to two genes (-length 2), and disabled the option for filtering only a certain number of highest scoring alignments (-filter 0). To account for the uncertainty of synteny breakpoints, we considered all SVs as polarized, if they overlapped syntenic regions as defined by cyntenator.

### Protein domain annotation and definition of homology relationships

We used the program hmmsearch (option -E 0.001) to search for known protein domains in the set of 30,884 predicted *P. pacificus* protein sequences (version TAU [24]), as defined by the PFAM database. The search program hmmsearch and the PFAM domain database were both obtained from the HMMER package (version 3.0).

In order to identify homologs for *P. pacificus* genes, we downloaded protein sequences for *C. elegans*, *C. briggsae*, *C. angaria*, *Haemonchus contortus*, *Meloidogyne hapla*, *Brugia malayi*, *Bursaphelenchelus xylophilus*, *Ascaris suum*, and *Trichinella spiralis* from Wormbase WS230 and *Heterorhabditis bacteriophora* sequences from Wormbase WS231. Furthermore we downloaded protein sequences for *Loa loa* and *Wuchereria bancrofti* from the filarial worms sequencing project, Broad Institute of Harvard and MIT (http://www.broadinstitute.org), *Meloidogyne incognita* protein sequences from the the *M. incognita* resources website (http://www6.inra.fr/meloidogyne_incognita), *Panagrellus redivivus* sequences from a website provided by Jagan Srinivasan, and protein sequences for *Dirofilaria immitis* from nematodes.org. Homologs for *P. pacificus* were identified by searching these data sets for BLASTP (version 2.2.28+) hits with e-value < 0.001. This resulted in 20,999 (68%) *P. pacficus* proteins with homologs in other nematode species and 9885 (32%) *P. pacficus* proteins without homologs (orphan genes).

We predicted one-to-one pairs between *P. pacificus* and *C. elegans* by using a variant of the widely employed methodology of best-reciprocal hits [24, 31, 32]. More precisely, we first defined inparalogs and then assigned best-reciprocal hits as one-to-one orthologs, only if neither the *C. elegans* nor the *P. pacificus* protein had any inparalog. Hereby, inparalogs were defined by following a similar methodology as implemented in the Inparanoid method [33], i.e. by identifying intraspecies BLASTP pairs that are more closely related than the best inter-species pairs. This procedure predicted 5985 one-to-one orthologous pairs.

The set of *P. pacificus* orphan genes and genes with homologs in other nematode species (excluding *P. pacificus* one-to-one orthologs with *C. elegans*), were further subdivided into singleton sequences and genes with putative paralogs by computing an adjacency matrix out of BLASTP hits within *P. pacificus* and extracting all connected components. Proteins that were members of connected components of size greater than one, were classified as “with paralogs”, or “singletons” otherwise. In the case of genes with homologs in other nematode species, the category “with paralogs” (*N* = 11,919) represents genes with many-to-many and many-to-one relationships, while singletons (*N* = 3095) represent one-to-many orthology relationships. Similarly, the 9885 *P. pacificus* orphan genes were divided into 4820 genes with paralogs and 5065 singletons.

### Population genomic analysis

Variants for population genomic analysis and diversity estimates in 100kb windows were obtained from the previously mentioned data set [12]. The phylogenetic tree in Fig 1A was generated by picking representative strains capturing roughly the global diversity of the sequenced 104 strains [12] and extracting variable sites that were sufficiently covered in all strains and in whole genome alignments with the outgroup *P. exspectatus*. To avoid long branch attraction effects we have only used sites where both alleles are shared between at least two of the samples, ignoring strain specific variation and fixed changes between *P. pacificus* and *P. exspectatus* since their separation. The resulting 457,097 parsimony informative sites were used to generate a neighbor joining tree on Hamming distances. The exclusion of singletons from the data set leads to a vast underestimation of distances, yet allows for a more robust estimation of overall tree topology.

Candidate sites that segregate between duplicate copies were extracted by filtering samtools v0.1–18 variants for sites with FQ score > 0 and quality score ≥ 20. Such apparent heterozygous sites allow to distinguish the two duplicated copies as the ambiguity in base calls is to a large extent due to misaligned divergent reads from one of the copies [12]. In this study, we use the term ‘alleles’ to denote two non-identical nucleotides that distinguish the two copies and for which resequencing data is mapped to the same position in the *P. pacificus* reference assembly. Allele counts from the RNA-seq and genomic alignments were obtained by building pileup files for the selected positions using the samtools (0.1.18) mpileup program (options: -A -q 2) [21] and extracting the relevant allele counts using custom perl scripts.

## Results

In order to investigate gene duplication and losses between different natural isolates of the nematode *P. pacifucs*, we decided to focus on two isolates RS5410 and RS5200, for which genome [12] and transcriptome data [13] is available. RS5410 is a *P. pacificus* strains that was sampled on la Reunion Island, whereas RS5200 was found in India. Both strains are members of different *P. pacificus* clades and show ∼ 1% genetic diversity when compared to each other, as well as with respect to the reference strain PS312 [12]. Fig 1A shows a general overview of the phylogenetic relationships between the analysed strains and the outgroup *P. exspectatus*.

We identified large deletions and duplications in RS5200 and RS5410 based on read coverage comparison with a reference strain (PS312). We optimized the predictions of SVs using an empirical data set of hundreds of manually classified SV predictions and chose cutoffs that showed a good tradeoff between true and false positives (Fig 1B). For the strain RS5200 this procedure resulted in 1621 and 609 predicted deletions and duplications, respectively, and 1642 and 565 predicted deletions and duplications for RS5410. Predicted SVs span a total range from two to 75kb. The portion of the *P. pacificus* genome that was predicted to be affected by deletions and duplications was 7.9Mb (4.6%) and 2.2Mb (1.3%) for RS5200 and 8.7Mb (5%) and 2.1Mb (1.2%) for RS5410. To rule out, that cnv-seq shows a tendency to predict deletions, rather than duplications, we tested RS5200 and RS5410 against each other showing very similar numbers of deletions and duplications, indicating that the higher number of predicted deletions is due to the fact, that we used PS312 as control sample.

We next tested, whether the genome of *P. pacificus* shows some hotspots of SVs. To this end, we divided the *P. pacificus* assembly into non-overlapping windows of 100kb size and plotted the fraction of a given genomic window that was predicted as being deleted and duplicated, across the six *P. pacificus* chromosomes (Fig 1C). Interestingly, we find two large regions spanning several hundred kilobases with extensive deletions for both strains on the X chromosome. These two blocks are located mostly outside of syntenic regions with the sister species *P. exspectatus*, potentially suggesting, that these regions might reflect recent lineage specific SVs in the reference strain PS312. Predicted duplications show a more even distribution across the chromosomes with only minor clusters on chrIV, chrII, and chrX (Fig 1C).

### SVs preferentially affect lowly expressed genes

When assessing the effect of SVs on genes, we have to take into account that the breakpoints of SVs predicted by read coverage approaches are not well defined. In addition, we still expect, a certain fraction of false positive predictions (< 8%, see *Methods*). For this reason, we considered only genes as being deleted or duplicated with respect to the reference strain PS312, if the complete gene from first to last exon is covered by the SV. Following this definition we find 1240 and 322 genes as being deleted and duplicated, respectively, for RS5200 and 1397 and 289 genes as being deleted and duplicated for RS5410. 65 of the duplicated genes and 732 of the deleted genes were predicted in both strains suggesting that these events predate the split of the two strains and would correspond to single evolutionary events.

**Fig 2:**
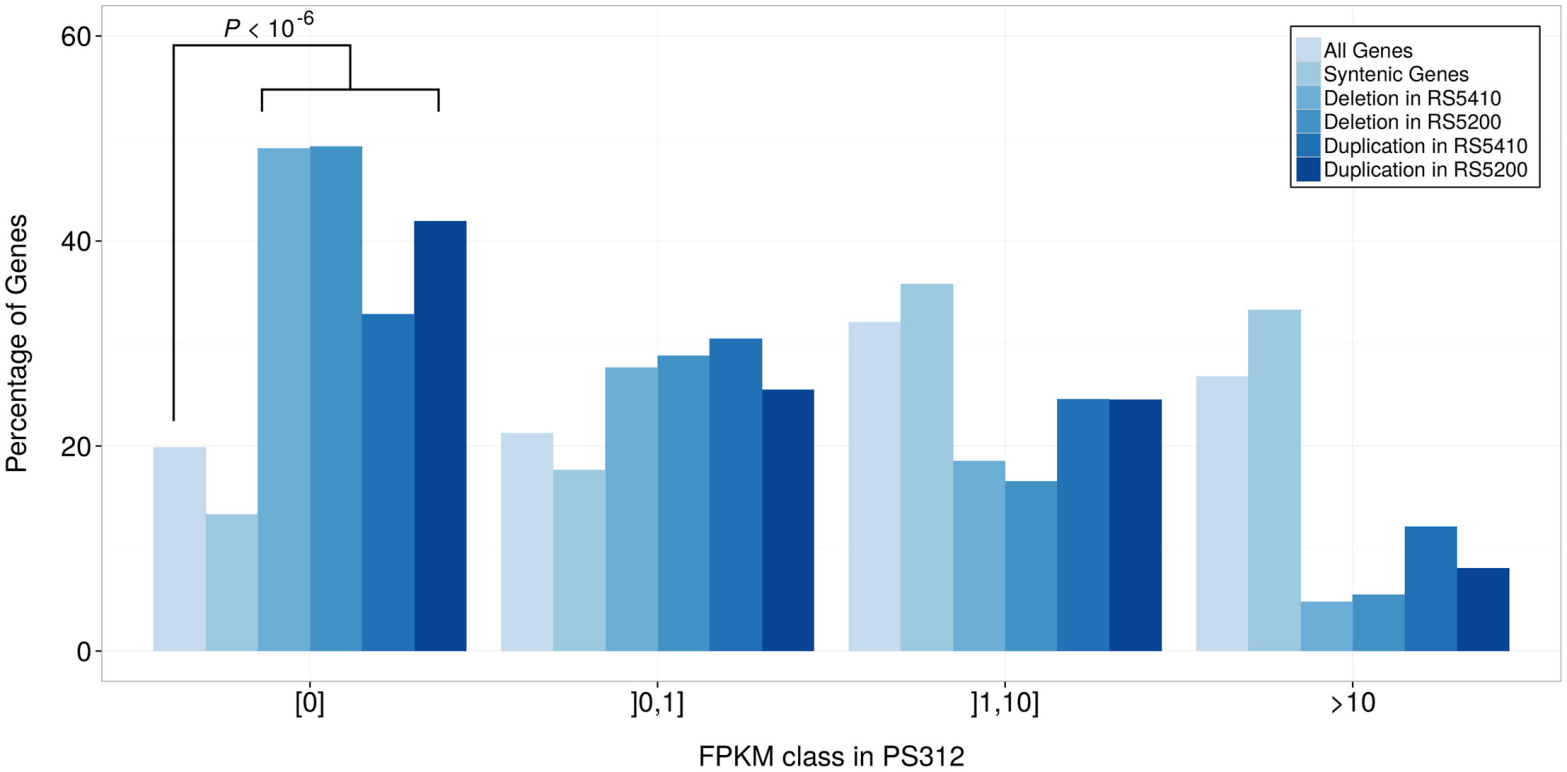
Expression levels of genes affected by SVs. The graphs show the distribution of all genes, syntenic genes, and genes that are affected by SVs across different expression classes in the reference strain PS312. The expression classes were obtained by dividing expression values in FPKM into classes that roughly span different orders of magnitude. An FPKM value of zero indicates the complete lack of expression evidence for such a gene. While syntenic genes are biased towards more highly expressed genes, genes in regions that are prone to SVs generally show significantly lower expression.

To further characterize the genes that are affected by SVs, we compared their expression levels in the reference strain PS312, where by definition all of them should be present. With respect to expression in the reference strain, we see a strong bias in genes with predicted deletions and duplications, i.e. they show a strong trend towards lower and even no expression (Fig 2).

Interestingly, with respect to differential gene expression analysis, this finding indicates, that most gene loss and gain events will not result in significant differential expression calls because of the low statistical power to detect significant differences for genes with few read counts. To test this, we carried out differential expression analysis [23] and found that only 25 and 24 (RS5410 and RS5200 respectively) deleted and 7 and 3 (RS5410 and RS5200 respectively) duplicated genes are identified as being significantly differentially expressed (FDR < 0.01). Also due to the lower expression of affected genes, we do not expect SVs to dramatically effect global transcriptome profiles as is demonstrated in the strong correlation (*ρ* > 0.89, Spearman) between expression values [13].

### Deletions and duplications show distinct transcriptomic readouts

**Fig 3:**
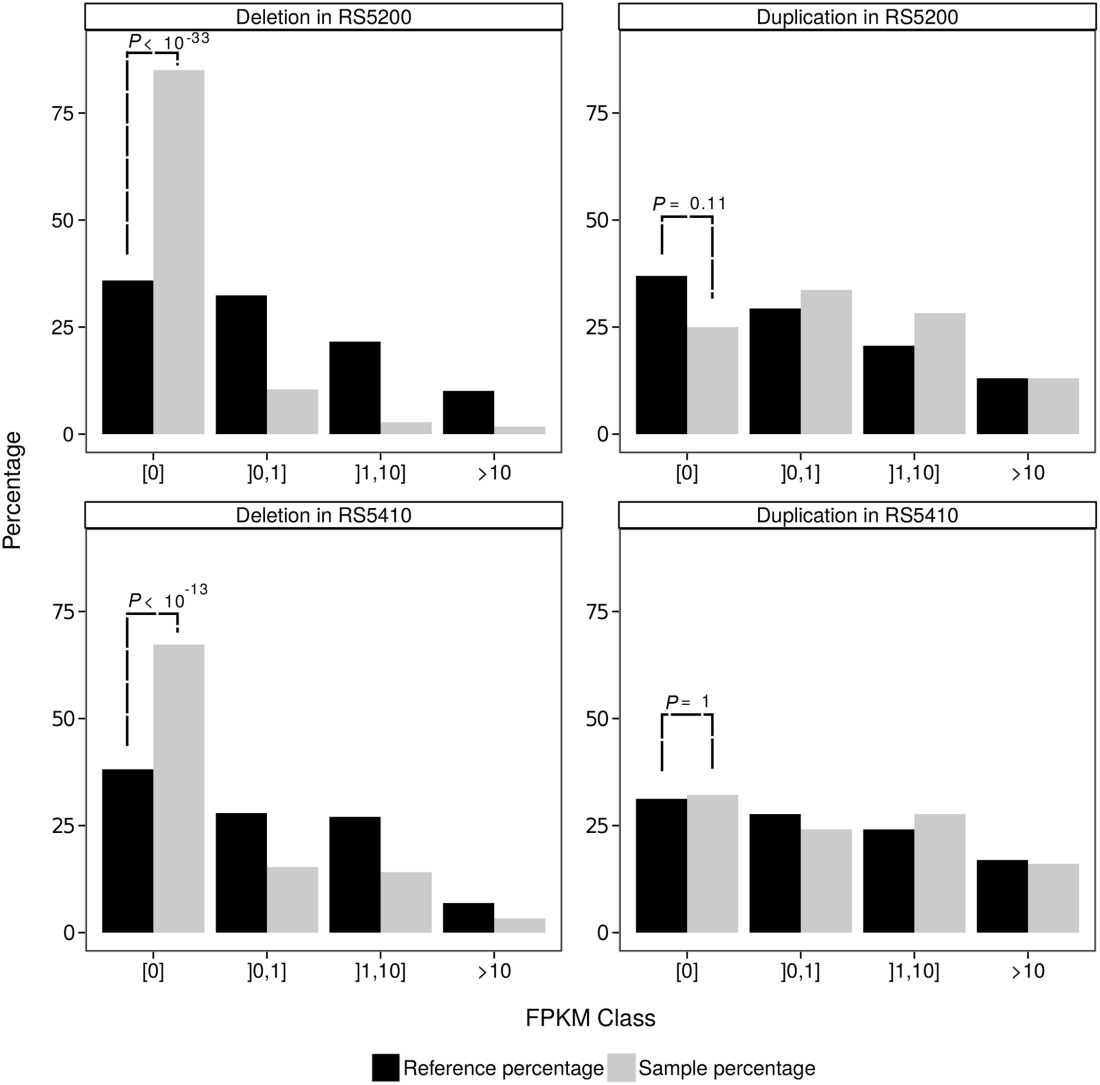
Effect of duplications and deletions on gene expression levels. Distribution of expression categories of duplicated and deleted genes for the reference strain and the strain of interest. Only genes within perfectly collinear regions between the reference genome and the sister species were used for this analysis. While deletions lead to a strong increase in genes without expression, i.e. more than 80% (RS5200) and 65% (RS5410) of genes in predicted deletions show indeed no evidence of expression. In contrast, we do not see the opposite trend for duplicated genes.

The previous analyses characterized genome-wide patterns of SVs in *P. pacificus*. However, to correctly interpret the identified genomic changes in an evolutionary sense, a polarization using an outgroup genome is needed. To this end, we made use of the recently assembled draft genome of the sister species *P. exspectatus* [12], which showed roughly 10% divergence to *P. pacificus* on the nucleotide level. Due to the inexact boundaries of predicted SVs, we decided to polarize the data by restricting the analysis to regions of perfectly collinear gene orders between the reference genome and the genome of the sister species (see Methods). We identified perfectly collinear regions spanning roughly 74.1Mb (43%) of the *P. pacificus* genome assembly. Within these regions, we considered all duplications and deletions as derived events and used this data to test the impact of SVs on gene expression levels. This polarization reduced the number of genes to 288 deleted and 92 duplicated genes for RS5200 and 333 deleted and 112 duplicated genes for RS5410. Fig 3 shows the distribution of expression levels in the reference strain PS312 and the two natural isolates RS5410 and RS5200 in classes, defined by predicted SV events. Despite the fact, that 35% of genes with predicted deletions in RS5200 show no expression even in the reference data, 80% of deleted genes show indeed no expression in RS5200 (*P* < 10^−34^, Fisher’s exact test). Similarly, we find a parallel trend in the strain RS5410 (*P* < 10^−14^, Fisher’s exact test). Given the various levels of uncertainty such as false positive predictions and the imprecise boundaries of SVs, which may explain evidence of expression in genes that are predicted to be deleted, these results strongly support that a large fraction of our predictions are indeed correct and are also reflected in terms of gene expression levels. To test, whether also partial deletions result in loss of expression, we checked if deletions that affect at least half of a given gene showed similar levels of lack of expression and we found in both strains a significant increase of genes without expression (*P* < 10^−6^, Fisher’s exact test). In contrast, when we compare the effect of predicted duplications on gene expression levels, we hardly see any signal towards higher expression of duplicated genes. Naively, we would expect two patterns, a higher fraction of genes with expression evidence (FPKM > 0) and a trend towards doubled gene dosage in the strain carrying the duplication. However, Fig 3 shows an insignificantly reduced fraction of genes (P = 0.11, Fisher’s exact test) without expression among duplicated genes in RS5200, yet this trend was missing in RS5410. In addition, comparing the fold changes of expressed genes (FPKM > 0) in both compared strains between duplicated genes and genes that are not affected by SVs, does not show any significant trend towards duplications leading to higher fold changes (*P* = 0.35 for RS5410 and *P* = 0.39 for RS5200, Wilcoxon-test).

This finding can be explained by the following three scenarios: i) The duplication does not encompass the whole gene including regulatory regions or the duplicated copy was inserted into a region where it is transcriptionally silent, ii) the higher dosage of the duplicated gene was compensated by mutations in cis-regulatory regions, iii) the duplicated gene is part of a highly self-regulatory network, that feeds back on its components and can thus compensate for the expression of the additional copy.

### Most duplicated genes show biased expression

In order to decide, which of the above mentioned scenarios most likely explains our data, we first tested, whether both copies are indeed expressed. If indeed, both copies are expressed at similar levels, this would indicate that the expression level of both copies must somehow be downregulated by one of the two compensatory mechanisms. To this end, we searched for segregating sites, that potentially distinguish between the two copies. Such sites were identified previously as they appeared as apparent heterozygous variants in whole genome sequencing data [12]. *P. pacificus* is a diploid species, which does not rule out the possibility of truly heterozygous sites. However all analysed strains were inbred for at least ten generations [12] indicating that most of the apparent heterozygous variants derive from sites that segregate between copies of recently duplicated regions and for which resequencing reads are aligned to a single position in the reference genome assembly. Please note, that we do not have experimental proof that these sites are truly segregating between the two copies, but this interpretation is strongly supported by previous findings, that apparent heterozygous sites are clustered in regions of extremely high coverage, that their presence does not decrease by the degree of inbreeding, and that admixed strains do not show elevated levels of heterozygosity [12].

**Fig 4:**
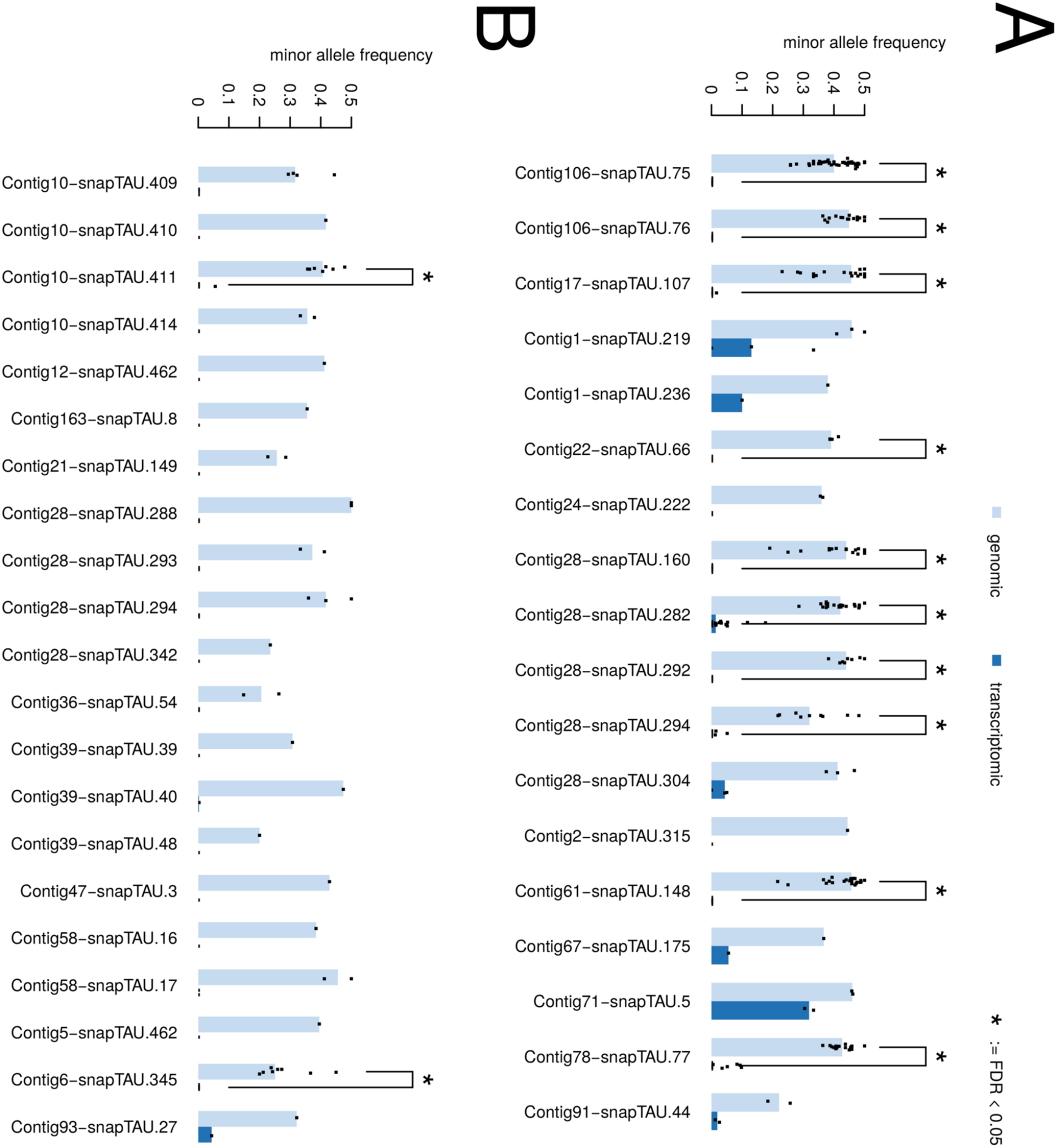
Biased allele expression in duplicated genes. For duplicated genes with putatively segregating mutations between the duplicates, we calculated the frequency of the minor allele in the genomic and transcriptomic alignments for all informative sites with coverage (≥ 10). Panel A shows the data for RS5200 and panel B for RS5410. The bars show median frequencies and the dots indicate the frequencies of all sites per gene. In twelve cases, we could identify a strong allele-specific bias between the genomic and transcriptomic data. This suggests that in the majority of examined cases the one of the duplicates is not expressed.

To test for the expression of both copies, we compared the ratios of segregating sites in the genomic resequencing and the transcriptome data. While we could only identify 45 informative sites in 21 genes with ≥ 10X coverage, in the genomic and transcriptome data for RS5410, we identified 199 sites in 18 genes, fulfilling these criteria for the RS5200 dataset. In the following, we will use the term ‘alleles’ to denote two non-identical nucleotides that distinguish the two copies. Fig 4 shows the distribution of minor allele frequencies for the genomic and transcriptomic data. In twelve cases, the difference between genomic and transcriptomic data proofed to be statistically significant (*P* < 0.05, Wilcoxon test with FDR correction). All these cases showed a bias of the minor allele not to be expressed. Similarly, in the majority of all tested genes the same trend is apparent indicating that the new copy is either not complete or was inserted into a region where it is transcriptionally silent. We interpret the fact that few genes showed expression of both alleles as evidence that in at least in some cases we were able to detect duplication events that produce a second functional copy. Intuitively, it is more likely that a deletion will have a functional effect on a gene rather than a duplication, because as we see, a duplication event must encompass the whole gene including promoter sequence and the insertion site must be in the right genomic context. To rule out that the lack of the expected upregulation is just an effect of partial duplications, we generated a set of predicted tandem duplications using the software pindel [29], which allows to identify structural variations at nucleotide resolution. However, based on 152 tandemly duplicated genes in RS5200 and 80 genes RS5410, we could neither detect a significant trend for more genes with expression evidence nor for higher fold changes when compared to unaffected genes.

The presence of segregating sites between duplicate copies suggests that the duplication events are generally older than duplication events without segregating sites. To test whether these two duplication events show different effect on gene expression, we performed tests for higher fraction of genes with expression (Fisher’s exact test) and for increased fold change (Wilcoxon test) but no significant difference could be identified between the two classes.

Finally, we would like to point out that the lack of expression evidence for the second copy is not an ultimate proof for its non-functionality. After duplication, the fate of the new genes could be pseudogenization, neofunctionalization, or subfunctionalization [34]. Gene expression domains can be highly stage-specific and in worms even cell-specific. Thus many functional genes with highly restricted expression patterns, which might be a result of subfunctionalization, are unlikely to be detected in RNA-seq data of pooled worms from various stages [13]. Furthermore, given the overall lower expression level of genes that were affected by SVs (Fig 2), it might well be that expression level differences of just one order of magnitude would result in the lack of evidence for the second copy.

### Evidence for negative selection and conservation of synteny

**Fig 5:**
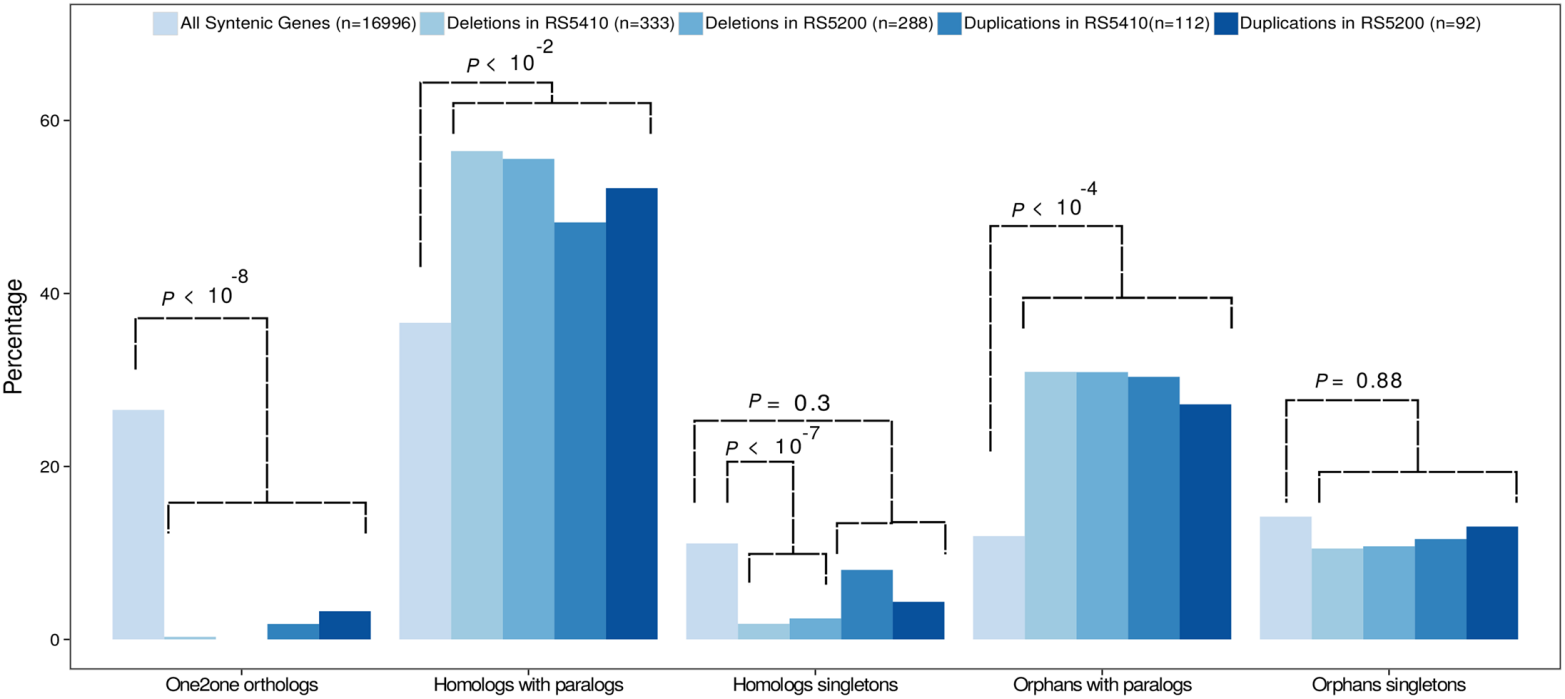
Depletion of SVs among highly conserved genes. We classified genes in deleted and duplicated regions (relative to the reference strain PS312) based on presence of protein domains as well as homology relationships with other nematodes including *C. elegans*. For the different homology classes, we find that predicted one-to-one orthologs with *C. elegans* are strongly depleted from SVs indicating the action of purifying selection. Conversely, conserved and orphan genes that are members of larger gene families are significantly enriched in SVs suggesting that similar processes that have generated different homology relationships at a cross-species level are operating on a microevolutionary level.

We next assessed the extent to which SVs affect gene classes as defined by different homology relationships with other nematodes (Fig 5). When compared to the overall distribution of gene classes, we find a strong depletion of deletions among single copy genes (one-to-one orthologs with *C. elegans* and genes with one-to-many relationships). On the contrary, multi-copy genes (many-to-X category, and orphans with paralogs) are found as being significantly enriched in SVs (Fig 5). The almost absence of one-to-one orthologs with *C. elegans* among SVs is expected, as the fact that these genes remained as single copies since the separation from their common ancestor, suggests a strong dosage sensitivity and consequently negative selection against SVs. Dosage sensitivity was proposed previously to cause selection against copy number variations in the human genome [17]. Given that a depletion of SVs among certain genes has already been observed in the human genome [17] and that we would expect a strong signature of negative selection based on previous population genomic analysis [12], this finding strongly supports the validity of a large part of our SV predictions. Interestingly, Fig 5 shows that one-to-one orthologs are not only are depleted among deletions but also among duplications. Under the presumption, that most of the duplications are not functional, this raises the question why they are nevertheless selected against. We interpret this finding as indirect evidence for selection to preserve synteny as it was shown that duplications tend to be local [35], leading to the possibility that insertion of duplicated sequences may interfere with long-range regulatory interactions or disrupt operon-like gene structures.

The absence of SVs in certain genomic regions could be explained if these genes were located in regions of peculiar properties such as low recombination rate. Since the available genetic linkage map of *P. pacificus* [36] does not provide sufficient resolution to test this, we use nucleotide diversity as an indirect measure of recombination. We have previously shown that diversity is reduced in gene dense regions [12], which is compatible with a model of background selection, i.e. in regions with low recombination frequencies, neutral variation will be selected against if it is linked to deleterious sites, leading to a reduction of diversity. Comparing the presence of SVs with nucleotide diversity in the most deeply sampled clade from Rödelsperger et al. [12], we find that 100kb windows without SVs show a strong reduction in nucleotide diversity in all comparisons. More precisely median diversity in regions without SVs is ten-fold lower than in regions with SVs, *P* < 10^−16^, Wilcoxon rank-sum test). This further supports the action of negative selection in these regions and suggests that even neutral SVs might be removed from populations due to background selection.

### Multiple gene families are enriched in SVs

**Fig 6:**
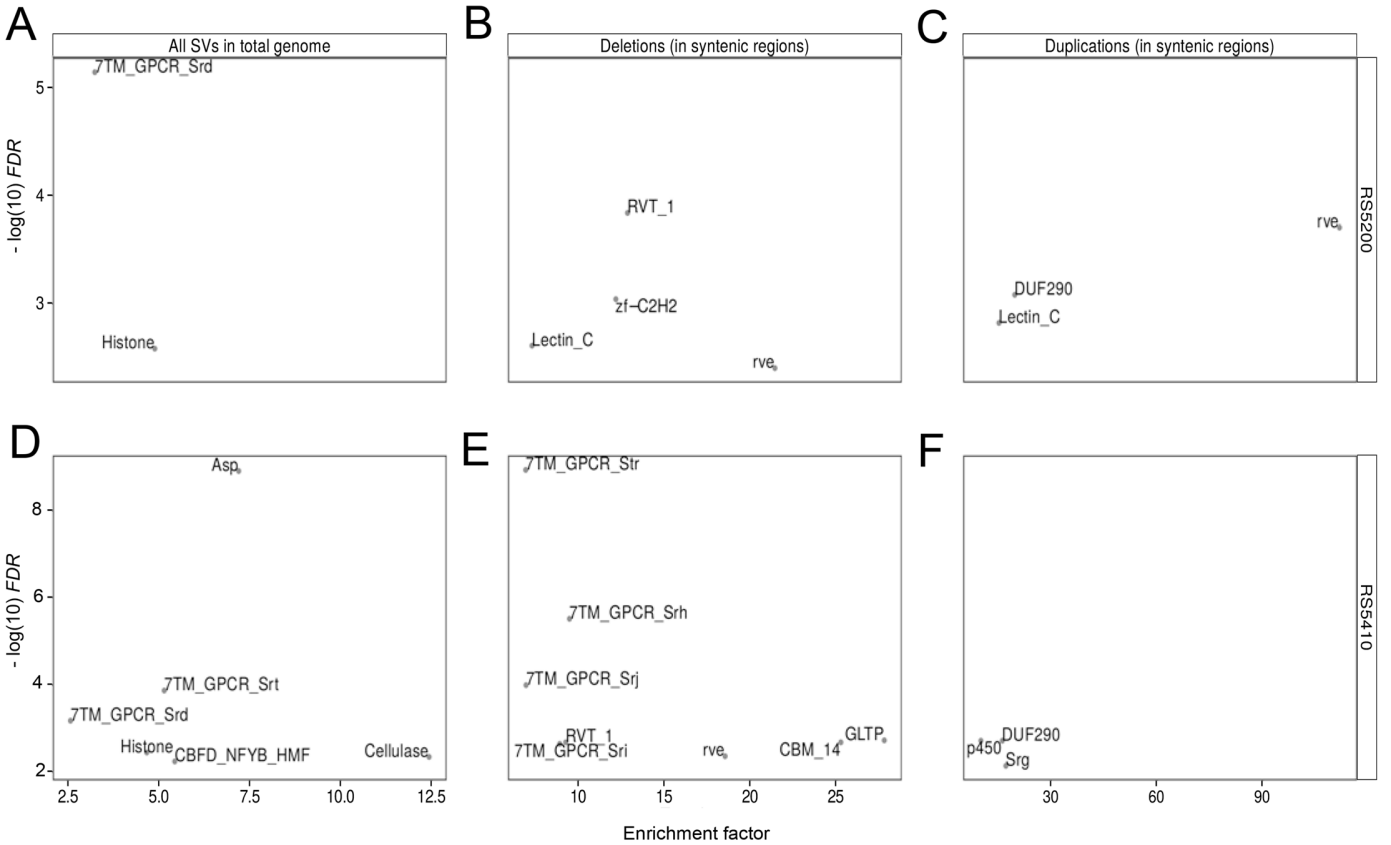
Gene families enriched in SVs. We defined gene families based on the presence of protein domains (PFAM) and tested whether genes of a given gene family are enriched in SVs. Panels A and D shows significantly enriched gene families for deletions and duplications relative to the reference strain PS312 as pooled data (unpolarized analysis). The other panels show the enriched gene families after restricting to perfectly collinear regions and interpreting the SVs as derived events (polarized analysis).

Finally, we identified gene families that show enrichment in duplications or deletions. This was done by defining gene families by the presence of a certain protein domain (PFAM) and then comparing how often members of a certain gene family were affected by different SVs relative to a random expectation (Fig 6). We carried out this analysis on the unpolarized SV data (pooled deletions and duplications with respect to the reference strain PS312) taking the whole genome as background set (Fig 6A, D) and then on the polarized data within regions of perfect gene collinearity between *P. pacificus* reference genome and the genome of *P. exspectatus*. For the strain RS5200, the most significant enrichment consisted in a strong overrepresentation of G-protein coupled receptors (GPCR, PF10317) among SVs. GPCRs comprise a family of 500–1000 genes in *C. elegans* that are known to play a role in sensing the chemical environment [37]. Similarly, the strain RS5410 shows a highly significant enrichment of GPCRs even in conserved syntenic regions (Fig 6E). Interestingly, GPCRs have also been previously identified to be enriched in copy number variations in *C. elegans* [9, 10]. Similarly, also C-type lectins (Fig 6C), which are known to play a role in immune response [38], have been described as enriched in copy number variations in *C. elegans* [9, 10]. These findings suggest that both gene families are actively evolving and are therefore prime candidates for genes playing a role in adaptation to new environments.

Another interesting gene family, that shows a significant enrichment in SVs in RS5410 are the cellulases (Fig 6D). The cellulales are the most prominent gene family of genes that were acquired by horizontal gene transfer [4, 26, 39]. Previously, seven cellulases were identified in the reference genome of *P. pacificus*. According to our data, RS5410 should have only three cellulase genes (4 deletions).

For RS5200 we also find a strong enrichment of retroviral integrase genes (rve, PF00665) among duplicated genes (Fig 6. C). The corresponding *P. pacificus* genes have no perfect matches in the NCBI nr database and the most similar sequences (≈ 40% identity) were from other nematodes such as *Caenorhabditis* and *Haemonchus contortus*. We did not find any literature about the function of this gene family outside of retroviruses and can only speculate that our finding may point towards recent activity of an unknown nematode-specific retrovirus.

Given that many different gene families show a strong tendency to be affected by SVs, we would like to point out that this might potentially affect our finding of lower gene expression of genes affected by SVs. It has been previously observed that the analysis of the impact of duplications on gene expression is complicated by the fact that non-uniquely mapping reads are generally discarded from RNA-seq data and similarly microarray probes are designed to be unique in the genome [40]. Thus it might be, that members of highly expanded gene families have an effectively smaller mappable transcript length than single copy genes, which can lead to a lower estimated expression level. However, given that the analysed RNA-seq data was sequenced as 100bp paired ends [13], we think that mappability only has a minor effect on gene expression estimates.

## Discussion

The question how genes are gained and lost during evolution is central to understanding the diversity across the animal kingdom. To complement previous comparisons of nematode gene repertoires at cross-species levels [4–6], we have investigated intra-species patterns of gene loss and gain in the nematode *P. pacificus*. Such comparisons spanning different time periods have the potential to illuminate different aspects of evolutionary forces acting on the *P. pacificus* genome. Along these lines, we have previously shown that mutations that occurred during one hundred generations under laboratory conditions show characteristics of neutral variation [41], whereas even in the most closely related natural isolates, we already observe strong evidence for purifying selection [12]. At the level of gene gain and loss, previous comparative genomic studies have highlighted massive gene contraction and expansion events that were hypothesized to reflect adaptation to new environments and lifestyles such as the necromenic association of *Pristionchus* nematodes with scarab beetles [4, 42]. However, at a population level, the prevailing pattern seems to be, that most deletions and duplications are either neutral or deleterious. Evidence for negative selection as shown in the strong depletion of SVs in large parts of the genome is consistent with previous evidence for purifying selection as obtained from single nucleotide variation data, such as the loss of nonsynonymous diversity over time [12].

Despite the fact that copy number variations and SVs have extensively been studied in various species [9–11, 17–19], only few studies have combined SVs with expression data [17–19]. Interestingly, we find that while deletions show a strong impact on gene expression, duplications have virtually no effect on the transcriptomic level. Based on the fact that most of the genes, that allowed to distinguish the different gene copies, showed a strong allelic bias in their expression, we hypothesize that the missing signal is due to either incomplete duplicates or duplicate insertions into transcriptionally silenced genomic regions. Further analysis of tandem duplications at nucleotide level resolution showed that even in cases where we can be sure that the duplication encompasses the first and last exon, we do not find the expected effect on expression data. This is in contrast to a previous study in humans [19], which showed a strong effect of duplications on gene expression levels. However, we would like to point out, that although in many cases, gene expression levels scaled linearly with copy number, only few cases truly showed that a duplication doubled the gene dosage [17, 19]. Furthermore, genetic diversity in *P. pacificus* is roughly one order of magnitude higher than in humans, suggesting that at the evolutionary distances between our *P. pacificus* strains, selection might have had more time to purge functional duplications that have slightly deleterious effects. Similarly, a recent gene duplication in *P. pacificus* which was identified by our depth of coverage approach showed significant differential expression and was present only in the reference (PS312) and it most closely related strain [27]. However, much more genomic and transcriptome data is needed to study the connection between age and effect of duplications on expression.

Intuitively, deletions are more likely to affect genes than duplications, because even a partial deletion may disrupt gene structure whereas a partial duplication most likely results in a nonfunctional gene fragment. Consistently, it has previously been shown that median size of recent duplications in *C. elegans* is shorter than average gene length (2.5kb) [35], further supporting the finding of abundant non-functional gene copies. One major drawback of our study is that using gene expression level to evaluate functionality is naive. However, in the absence of true functional data for most of the genes in *P. pacificus*, the use of gene expression as a first proxy for functionality revealed very interesting trends that might also be reflected at the functional level.

Despite the fact that most novel gene copies are not expressed at comparable level to their ancestral copy, an open question remains, why there seems to be strong selection against these apparent nonfunctional duplications in large parts of the genome. We can only speculate, that insertions of even non-functional duplicated sequences may interfere with long range regulatory interactions and may disrupt gene and operon-like structures. Previous analysis in *C. elegans* has shown that recent duplications tend to be locally and show a trend towards dispersal across the genomes with increasing age [1, 35]. Thus, the local nature of duplications suggests that in regions where synteny is important, even non-functional duplications may have deleterious effects. However, better sequencing data such as longer reads and higher coverage as well as more exact bioinformatics approaches are needed to precisely locate breakpoints of SVs and to further support the conclusions that we have drawn from the analysis of genomic and transcriptomic data for a limited number of natural isolates. Finally, the fact that they were generated and maintained may suggest that some of the duplications might be beneficial for certain strains. Thus, further studies could also address the adaptive potential of gene duplications in the evolution of *P. pacificus* nematodes.

## Conclusions

In this study, we have shown that read coverage approaches for identification of SVs provide sufficiently well predictions to gain first insights into the microevolution of gene gain and loss, but future work will need better data and methods to investigate SVs at a nucleotide level resolution. Our study reveals similar trends as were shown previously in humans [17], i.e. negative selection on gene dosage in large parts of the genome and similar to previous studies we do not find that duplications cause a doubled gene dosage [17, 19, 40]. Finally our study highlights that evolutionary comparisons at various time-scales are needed to get a full picture of mutational processes that may guide the adaptation to new environments and may help to better understand the diversity observed across all metazoan life.
